# Mitogenomics reveals high synteny and long evolutionary histories of sympatric cryptic nematode species

**DOI:** 10.1002/ece3.1975

**Published:** 2016-02-20

**Authors:** Tara Grosemans, Krystalynne Morris, William Kelley Thomas, Annelien Rigaux, Tom Moens, Sofie Derycke

**Affiliations:** ^1^ Marine Biology SectionBiology DepartmentFaculty of ScienceUniversity of GhentKrijgslaan 281 (S8)9000GentBelgium; ^2^ Department of Biochemistry and Molecular BiologyHubbard Center for Genome StudiesUniversity of New Hampshire35 Colovos RoadDurhamNew Hampshire03824; ^3^ CeMoFeUniversity of GhentKarel Lodewijk Ledeganckstraat 359000GentBelgium; ^4^ Royal Belgian Institute of Natural Sciences (RBINS)OD Taxonomy and PhylogenyVautierstraat 291000BrusselsBelgium

**Keywords:** Adaptation, cryptic speciation, *Litoditis marina*, Miocene, *Wolbachia*

## Abstract

Species with seemingly identical morphology but with distinct genetic differences are abundant in the marine environment and frequently co‐occur in the same habitat. Such cryptic species are typically delineated using a limited number of mitochondrial and/or nuclear marker genes, which do not yield information on gene order and gene content of the genomes under consideration. We used next‐generation sequencing to study the composition of the mitochondrial genomes of four sympatrically distributed cryptic species of the *Litoditis marina* species complex (PmI, PmII, PmIII, and PmIV). The ecology, biology, and natural occurrence of these four species are well known, but the evolutionary processes behind this cryptic speciation remain largely unknown. The gene order of the mitochondrial genomes of the four species was conserved, but differences in genome length, gene length, and codon usage were observed. The *atp8* gene was lacking in all four species. Phylogenetic analyses confirm that PmI and PmIV are sister species and that PmIII diverged earliest. The most recent common ancestor of the four cryptic species was estimated to have diverged 16 MYA. Synonymous mutations outnumbered nonsynonymous changes in all protein‐encoding genes, with the Complex IV genes (*coxI‐III*) experiencing the strongest purifying selection. Our mitogenomic results show that morphologically similar species can have long evolutionary histories and that PmIII has several differences in genetic makeup compared to the three other species, which may explain why it is better adapted to higher temperatures than the other species.

## Introduction

Species with similar morphologies but distinct genetic differences (= cryptic species) abound in all animal taxa and in all geographical areas and indicate that morphological stasis is a constant evolutionary phenomenon (Pfenninger and Schwenk 2007). Allopatric speciation has been the basic mechanism to explain species formation (Coyne and Orr 2004), even in marine environments where barriers to gene flow are less obvious, population sizes are large, and dispersal is substantial (Knowlton 1993; Palumbi 1994). Cryptic species have long been recognized in marine environments and are often found together (Knowlton 1993; Stuart et al. 2006). This may point to secondary contact of species that speciated allopatrically, or to the occurrence of species formation in sympatry (Via 2001). In the latter scenario, monophyletic sister clades are expected to occur in sympatry (Coyne and Orr 2004). The discovery of cryptic species is generally a side product of population genetic and phylogeographic studies, resulting in a delineation that is often based on a phylogeny generated with a limited number of loci. As such, alterations in gene order, content, and size that may occur during or after the speciation event remain undetected. Mitochondrial (mt) genomes of animals are particularly interesting to investigate evolutionary relationships between closely related species because they evolve faster than nuclear genomes, their small size, simple structure, absence of recombination, and high variability (Castellana et al. 2011).

The metazoan mt genome is typically characterized by a circular double‐stranded molecule – but exceptions have been observed, for example, in the nematode *Globodera pallida* where six small circular molecules were found (Armstrong et al. 2000; Gibson et al. 2007). It encodes for 2 rRNAs, 22 tRNAs, and 13 proteins that are essential for mitochondrial functioning (Boore 1999). The proteins are typically under strong purifying selection (Meiklejohn et al. 2007; Castellana et al. 2011) which prevents the accumulation of mutations that may alter the functional protein products of important subunits of the respiratory chain. Variability in metazoan mitochondrial gene content rarely involves protein‐coding and rRNA genes, and is mostly attributed to tRNA genes (Gissi et al. 2008). Phylogenetic relationships based on mitochondrial DNA can be blurred by the presence of *Wolbachia* which can cause introgression and/or selective sweeps (Hurst and Jiggins 2005). Nevertheless, a mitogenomic approach has yielded novel insights in phylogenetic relationships of many organisms, including nematodes (Sun et al. 2014; Zasada et al. 2014).

Nematodes form one of the most successful animal phyla in terms of species diversity and habitat exploitation (Bongers and Ferris 1999). Substantial cryptic diversity has been observed in parasitic (de León and Nadler 2010), freshwater (Ristau et al. 2013), and marine species (Derycke et al. 2013). The genetic differences underlying this cryptic speciation have however been restricted to the use of a few loci (the mitochondrial cytochrome oxidase c subunit 1 (*coxI*) gene, and fragments of the nuclear ribosomal units). Population genetic and phylogeographic studies have demonstrated the presence of at least 10 cryptic species within the *Litoditis marina* morphospecies – formerly known as *Rhabditis (Pellioditis) marina* (Bastian 1865) – in the northeast Atlantic (Derycke et al. 2008a,b). Increasing sympatry was observed when species were more distantly related, suggesting a predominant mode of allopatric speciation (Derycke et al. 2008a,b). Four of these species (PmI, PmII, PmIII, and PmIV) co‐occur on decomposing macroalgae in the littoral zone of coastal and estuarine environments in Belgium and the Netherlands (Derycke et al. 2005). The four species lack diagnostic morphological features and are reproductively isolated (Fonseca et al. 2008). Temporal fluctuations in their distribution have been observed (Derycke et al. 2006). In addition, differences in competitive ability (De Meester et al. 2011), timing of dispersal (De Meester et al. 2012), and microbiomes (Derycke et al. submitted) have been demonstrated. Although their life histories and ecological interactions are beginning to be unraveled, the evolutionary history of this species complex remains largely unknown. Phylogenetic relationships based on the nuclear ribosomal ITS and 28S regions showed that PmI and PmIV are sister taxa, which are more closely related to PmII and more distantly related to PmIII (Derycke et al. 2005, 2008a,b). Although the four species were recovered in the mitochondrial COI gene phylogeny, deeper relationships were not supported (Derycke et al. 2008a,b). In this study, we investigated the whole mitochondrial genomes of these four sympatrically distributed cryptic species and specifically aimed at identifying differences in genomic architecture (size, gene order, codon usage) between the four species. In general, high synteny and chromosomal organization are observed between rhabditid species (Nigon and Dougherty 1949; Hillier et al. 2007; Bik et al. 2012; Sun et al. 2014), although *Caenorhabditis briggsae*, the cryptic sister species of *C. elegans*, shows some unusual *nad5* pseudogenes and associated heteroplasmic deletion events that suggest a dynamic evolution between *C. elegans* and *C. briggsae* (Howe and Denver 2008; Phillips et al. 2015). Second, we investigated phylogenetic relationships using all mitochondrial protein‐coding genes. Based on the phylogenetic relationships established from two nuclear loci, we expected PmIII to be most distantly related to the other species and to recover PmI and PmIV as sister taxa. Third, we estimated the timing of divergence between the four species. Phylogeographic results have suggested that this timing was before the last glacial maxima in the Pleistocene (Derycke et al. 2008a,b), but finer time estimates are lacking. Finally, we explored selective pressures on the protein‐coding genes (PCGs), and we expected to observe purifying selection because of the important functions of mitochondrial protein‐coding genes in the respiratory chain.

## Material and Methods

### Nematode cultures

Four monospecific cultures of *Litoditis marina* were kept in the lab under stable conditions at 18°C. Species PmI, PmII, and PmIII were isolated from *Fucus* fragments from Paulina (51°21′N, 3°49′E), a saltmarsh in the polyhaline area of the Westerschelde estuary (the Netherlands). Species PmIV was isolated from Lake Grevelingen (51°44′N, 3°57′E), a marine lake in the Netherlands. Each culture was started from a single female and was maintained in the lab for many generations before the experiment. Nematodes were cultured on marine agar plates (25 psu, 4/1 bacto/nutrient agar in a final concentration of 1%) to which 2 mL of Tris‐HCl (pH 8) was added to buffer the pH of the plates, and which were seeded with *Escherichia coli* K12 as a food source. All females were morphologically identified using a stereomicroscope and the species description of *Rhabditis (Pellioditis) marina* (Bastian, 1865) as outlined in (Inglis and Coles 1961). Species identity of the cultures was checked via qPCR (Derycke et al. 2012). After successful establishment of the cultures, DNA was extracted from a single nematode by transferring the worm to a 0.5‐ml Eppendorf tube containing 20 *μ*L worm lysis buffer (50 mmol·L KCl, 10 mmol·L Tris pH 8.3, 2.5 mmol·L MgCl2, 0.45% NP 40, 0.45% Tween‐20). Tubes were frozen at −20°C to disrupt cuticula and cell membranes, after which 1 *μ*L of proteinase K (10 mg·ml^−1^) was added. Lysis was performed by incubating the tubes for 1 h at 65°C and 10 min at 95°C. DNA samples were centrifuged for 1 min at 20,800 g and were then used as template for qPCR.

Assignment of nematodes to one of the four cryptic *L. marina* species were performed with qPCR using species‐specific primers located in the ribosomal internal transcribed spacer (ITS) region (Derycke et al. 2012). The qPCRs were prepared in 10 *μ*L volume containing 5 *μ*L SensiMix SYBR No‐ROX One‐Step (2×) solution, 3 *μ*L of each primer (final concentrations of 1 *μ*mol·L for Pm I and Pm III, 500 nmol·L for Pm II, and 200 nmol·L for Pm IV (Derycke et al. 2012)), 1 *μ*L PCR‐grade water, and 1 *μ*L of DNA template. The thermal cycling protocol consisted of an initial denaturation for 10 min at 95°C followed by 40 cycles of denaturation for 10 s at 95°C, annealing for 20 s at 60°C, and extension for 20 s at 72°C. All specimens were analyzed with all four primers, and the primer set yielding a positive signal was used to identify the specimen as PmI, PmII, PmIII, or PmIV.

### DNA extraction and next‐generation sequencing

Nematodes were removed from the agar dishes by sucrose washing (using sucrose in a final concentration of 40%) and washed four times in artificial seawater (ASW). DNA was extracted from several hundred nematodes of each species using the CTAB protocol as described in (Derycke et al. 2012). DNA concentrations were measured with a Nanodrop ND2000, and quality of the DNA was checked by gel electrophoresis before sending the samples to the Hubbard Center for Genome Studies (University of New Hampshire, USA). Sequencing libraries for the four samples were generated following the “Low Throughput Sample Protocol” for Illumina TruSeq DNA libraries. Approximately 1 *μ*g of DNA was sheared by ultrasonification using a Covaris M22 to a target size of 500 bp. The overhangs resulting from the shearing were converted to blunt ends using the End Repair Mix of the Illumina kit. The resulting samples were cleaned with AMPure Beads, after which a single “A” nucleotide was added to the 3′ ends of the fragments using the A‐Tailing mix of the Illumina kit. For each individual species, a unique adaptor/index was ligated to the DNA fragments after which the samples were cleaned with AMPure Beads. The ligation products were then loaded on a 2% agarose gel to remove unligated adapters and adapter dimers and to size‐select the fragments at 500 base pairs. Samples were then purified with the MinElute Gel Extraction Kit (Qiagen, Benelux, Antwerp, Belgium). DNA fragments with adapters on both ends were then enriched by PCR with primers that anneal to the ends of the adapters and using an initial denaturation of 98°C for 30 s, 10 cycles of 98°C for 10 s, 60°C for 30 s and 72°C for 30 s, and a final extension of 5 min at 72°C. PCR products were cleaned with AMPure Beads and analyzed for size and concentration on an Agilent Bioanalyzer. The four libraries were quantified through qPCR (Kapa Biosystems), pooled, and loaded on two lanes of a single rapid‐run flow cell for paired‐end sequencing (2*150 bp) on the Illumina HiSeq 2500.

### Data analysis

#### Assembly and annotation of the mtDNA

A de novo assembly was generated with the CLC main workbench software (www.clcbio.com). As we were mainly interested in the mitochondrial DNA, we filtered the results table containing the contigs from the assembly, with filters set on consensus length >230 and total read count >5. *Litoditis marina* is a rhabditid nematode and belongs to the same family as the model organism *Caenorhabditis elegans* for which a completely annotated genome is available. The mitochondrial genome of *C. elegans* was downloaded from Genbank (accession number NC_001328.1). A local BLASTn search was performed with the mtDNA of *C. elegans* as query, using default settings except for the e‐value, which was set at 1e‐10, and the number of threads, which was set at 50. From the retrieved contigs, those in the size range of mtDNA (13,000 bp) were retained for further analyses. GC content for the mtDNA was calculated in R 3.0.1 (http://www.r-project.org/) using the seqinR package (Charif and Lobry 2007).

Open reading frames (ORFs) in the contigs containing the mitochondrial genomes were searched using CLC. Annotation of the ORFs was performed using a BLASTn search against Genbank and with e‐value set at 1e‐10 and specified for Metazoa. CLC detected 11 ORFs and the lacking protein‐coding gene, *nad4L*, was found by a specific query using the protein‐coding gene sequence of *C. elegans* (extracted from the complete mt genome of *C. elegans* accession number NC_001328.1) against our mtDNA contig. Once the 12 expected ORFs were annotated, they were verified by checking the start and stop codon positions and by translating the sequence to protein. For all genes, pairwise distances were calculated (p‐distribution model, pairwise deletion) between all four species in MEGA6 (Tamura et al. 2013). Codon usage was calculated for the four species in MEGA6 and the Relative Synonymous Codon Usage (RSCU) (Sharp et al. 1988) values were used to compare between species. Gene density of the mitochondrial genomes was calculated by dividing the number of genes by the genome size. The genome size was determined with exclusion of the AT‐rich region because assembly algorithms generally cannot assemble completely through the AT‐rich regions and there may be numerous small contigs for this region that are not included in the main contig. Consequently, variability in length may be caused by an inadequate assembly rather than by actual differences in genome size between the species.

tRNA detection was performed in several steps. First, all the tRNA sequences were extracted from the *C. elegans* complete mt genome (accession number NC_001328.1) and then queried via BLASTn against the mtDNA of the four cryptic species to find their positions. The secondary structure of these tRNAs was determined in CLC, and we checked that the anticodon was located in the loop (further referred to as manual procedure). Second, the mtDNA was uploaded in tRNAscan‐SE 1.21 (Lowe and Eddy 1997) to verify the detected tRNA, with source settings on Nematode Mito. Only the two genes for tRNA^Ser^ were not confirmed with tRNAscan‐SE. Finally, the mtDNA sequence was also imported in ARWEN (Laslett and Canbäck 2008), another tRNA detection tool, in a further attempt to identify the tRNA^Ser^ not identified with the previous algorithms. With ARWEN one of the two tRNA^Ser^ could be identified.

The annotated mitochondrial genomes have been submitted to Genbank under Accession numbers KR815450 for PmI, KR815451 for PmII, KR815452 for PmIII, and KR815453 for PmIV.

#### Mode of selection on PCG

Synonymous (dS) and nonsynonymous (dN) substitution rates were calculated in MEGA6 (Tamura et al. 2013), using the Nei–Gojobori method and 500 bootstraps. When dN < dS, purifying selection eliminates new variants. When dN > dS, new variants are selected, and positive selection is acting. Under neutral evolution, dS is equal to dN. Afterward, a *Z*‐test of selection was performed as implemented in MEGA6, with the hypothesis tested being purifying selection, as dN < dS, and following settings were used: 500 bootstraps and the Nei–Gojobori method for synonymous–nonsynonymous substitution type.

#### Phylogenetic relationships and timing of divergence

All PCG sequences of the four species were extracted, translated to amino acids and individually aligned in CLC, including *C. elegans* and *C. briggsae* homologs (downloaded from GenBank, NC_001328.1 and NC_009885.1). The aligned amino acid sequences of the PCGs were then untranslated to nucleotide sequences and concatenated into one fasta file, which was used to determine phylogenetic relationships in MEGA6 (Tamura et al. 2013). Neighbor‐joining settings included 500 bootstrap replications, the uncorrected p‐distance model and pairwise deletion of gaps, Maximum likelihood settings were GTR + G + I model and 500 bootstrap replications, and Maximum Parsimony settings were 500 bootstrap replications. Default settings were used for the other parameters. The evolutionary model that best fitted our data was obtained using default settings and treating gaps/missing data with partial deletion in MEGA6.

Additionally, the most recent common ancestor (MRCA) was calculated with BEAST v2.1.1, containing the BEAST, BEAUti, and TreeAnnotator programs (Bouckaert et al. 2014). Only the PCGs were used to make the alignment, which was then imported in BEAUti as a nexus file to be able to set the model parameters for BEAST. At the site models tab, the Gamma Category Count was set at 4, the Shape and Substitution rate was estimated and the GTR model was chosen as the substitution model (see above) with an empirical frequency. A normal relaxed clock model was selected, and the priors were set at the calibrated Yule model, and an extra criterion was added to define the calibration node. Divergence time of *C. elegans* and *C. briggsae* has been estimated at 18 MYA (Cutter 2008), and both species were set to be monophyletic within *Caenorhabditis* from this time point by defining a normal distribution with parameters (18, 0.5). The Markov Chain Monte Carlo settings were set at a chain length of 1,000,000, a trace log of 200 and screen log of 1000. TreeAnnotator was used to obtain an estimate of the phylogenetic tree. A 1% burnin was specified (being 50), the posterior probability limit was set at zero, and mean heights were chosen for the nodes. The generated tree file was visualized in FigTree v1.4.0. A second calibration point with the divergence between Chromadorea/Enoplea between 532 and 383 MYA (Rota‐Stabelli et al. 2013) was used for comparison.

### *Wolbachia* detection

The assembled contigs of the four species were searched for the presence of the endosymbiotic bacteria *Wolbachia* spp. Five characteristic genes (*coxA*,* gatB*,* hcpA*,* ftsZ*, and *fbpA*), the *wsp* gene, and the 16s rDNA gene (Doudoumis et al. 2012) of *Wolbachia* endosymbionts that have been found in the parasitic nematodes *Brugia malayi* and *Onchocerca* spp. were downloaded from Genbank (respective accession numbers for *coxA* are DQ842273 and FJ390245; for *gatB* DQ842421 and JX075229; for *hcpA* DQ842384; for *ftsZ* AY583309 and AJ276501; for *fbpA* DQ842347 and JX075225; for *Wsp* AY527201 and AY095210 and CU062443; and for the 16S rDNA AF051145 and AF172401). These sequences were queried against our contig databases containing all assembled contigs using BLASTn. A match was considered when the % identity score was higher than 80.

## Results

Total reads per species varied between 41 and 53 million and had a length of 151 bp (Table 1). The N50 lengths ranged from 1034 to 3017, and maximum contig lengths were more variable between species (PmI: 1,333,912; PmII: 1,570,033; PmIII: 1,838,899; and PmIV: 1,001,282).

**Table 1:** Summary of the Illumina HiSeq data. The four cryptic species of the *Litoditis marina* complex are indicated as PmI, PmII, PmIII, and PmIV

	PmI	PmII	PmIII	PmIV
N50	3117	1034	1890	1661
Total reads	52,067,680	41,399,444	52,823,308	42,346,932
Matched Reads	49,663,612	37,036,661	50,569,428	40,103,528
Number of Contigs	278,752	381,176	292,112	263,052
Maximum Contig Length	13,33,912	1,570,033	18,38,899	1,001,282
Average length contigs	1136	768	961	987
GC content (in %)	47.6	44.6	42.4	42.7

### Comparison of the *Litoditis* mtDNA genomes

**Figure 1:**
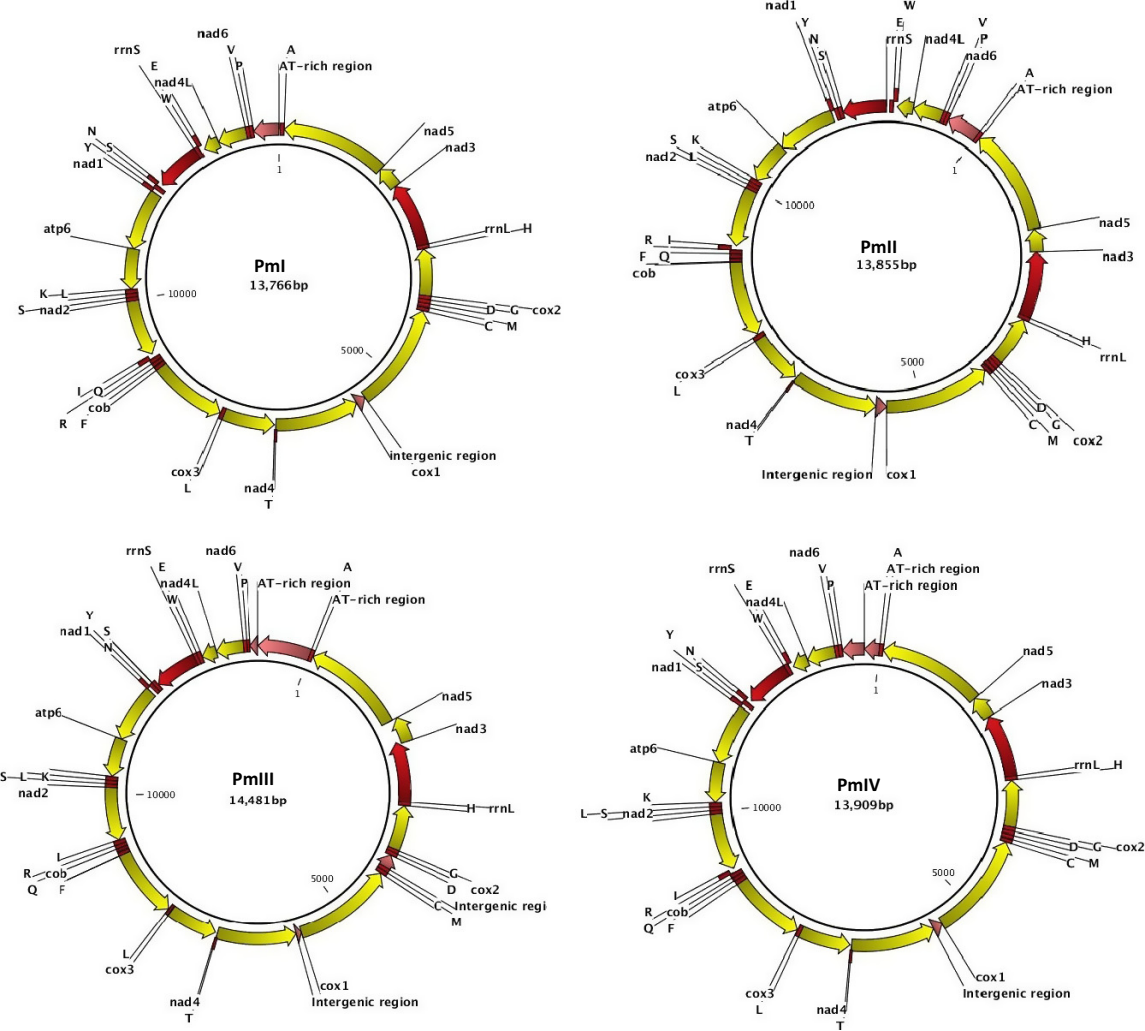
Mitochondrial genome of the four cryptic *Litoditis* species. All 12 protein‐coding genes are shown in yellow arrows, 2 ribosomal RNAs in red arrows, and the 22 transfer RNAs as red blocks. tRNAs are indicated by their single letter amino acid code. The 2 noncoding regions (AT‐rich region and the intergenic region) are shown in pink.

The mtDNA of each species was located on a single contig and was 13,766, 13,855, 14,481, and 13,909 bp long, for PmI, PmII, PmIII, and PmIV, respectively (Table 2). The mitochondrial genomes encode for 2 rRNAs, 22 tRNAs, and 12 proteins, which were positioned in the same order in each of the four *L. marina* species (Fig. 1). The assembled AT‐rich region showed considerable length variability between the four species and was considerably longer in PmIII (Table 2). The mitochondrial genome size excluding the AT‐rich region showed small differences in length between the four species, with the mt genome of PmIII still being 140–162 bp longer than that of the other species (Table 2). GC content was very similar between the four species and varied between 20.4 and 21.3%. PmIII had a somewhat lower percentage of coding information compared to the three other species (97.5% vs >98.2%) which was caused by the additional DNA sequence found in the intergenic region located between tRNA^Met^ and tRNA^Asp^.

**Table 2:** mtDNA characteristics of the four cryptic species (PmI, PmII, PmIII, and PmIV). Gene density was calculated by dividing the number of genes by the genome size without the AT‐rich region

	PmI	PmII	PmIII	PmIV
Genome size (bp)	13,766	13,855	14,481	13,909
AT‐rich region (bp)	378	479	953	543
Genome size (bp) without AT‐rich region	13,388	13,376	13,528	13,366
GC content (%)	21.2	20.4	20.6	21.3
Coding regions				
Proportion of the genome (%)	98.5	98.2	97.5	98.7
No. genes	36	36	36	36
Gene density (gene/kb)	2.7	2.7	2.7	2.7
Noncoding regions				
Proportion of the genome (%)	0.4	0.7	0.3	0.2
Intergenic regions				
Proportion of the genome (%)	1.1	1.1	2.2	1.1

### Protein‐coding genes (PCGs)

The gene order was the same in the four species (Fig. 1) and introns were absent in the PCG. Gene sizes of PmI and PmIV were identical, while *Atp6* and *nad4L* were one amino acid (AA) longer and shorter, respectively in PmII. In PmIII, *nad5* and *coxIII* were 1 AA longer than in the other three species and *nad3* was 13 AA longer (Table 3). The ATP synthase F0 subunit 8 (*atp8*) gene was lacking in the mitochondrial genomes of all four cryptic species.

**Table 3:** Length and position of the 12 PCGs, 2 rRNAs, and 22 tRNAs in the mitochondrial genomes of the four cryptic *Litoditis marina* species

		PmI				PmII				PmIII				PmIV			
		Position (nt)		Length (nt)	Start/Stop codon or anticodon	Position (nt)		Length (nt)	Start/Stop codon or anticodon	Position (nt)		Length (nt)	Start/Stop codon or anticodon	Position (nt)		Length (nt)	Start/Stop codon or anticodon
tRNA	Ala	22	77	56	TGC	1421	1476	56	TGC	834	889	56	TGC	228	283	56	TGC
Gene	nad5	79	1662	1584	ATT/TAA	1477	3060	1584	ATT/TAA	892	2478	1587	ATG/TAA	286	1869	1584	ATT/TAA
Gene	nad3	1663	1998	336	ATT/TAA	3063	3398	336	ATT/TAA	2481	2855	375	ATT/TAA	1870	2205	336	ATT/TAA
rRNA	rrnL	2000	2958	959		3400	4359	960		2818	3773	956		2207	3165	959	
tRNA	His	2959	3013	55	GTG	4360	4415	56	GTG	3774	3830	57	GTG	3166	3220	55	GTG
Gene	coxII	3017	3712	696	ATT/TAA	4419	5114	696	ATT/TAA	3834	4529	696	ATA/TAA	3224	3919	696	ATA/TAA
tRNA	Gly	3713	3767	55	TCC	5115	5170	56	TCC	4530	4584	55	TCC	3920	3974	55	TCC
tRNA	Asp	3768	3822	55	GTC	5171	5226	56	GTC	4585	4639	55	GTC	3975	4029	55	GTC
Intergenic region										4640	4845	206					
tRNA	Met	3823	3882	60	CAT	5227	5286	60	CAT	4846	4905	60	CAT	4030	4089	60	CAT
tRNA	Cys	3884	3941	58	GCA	5289	5345	57	GCA	4906	4962	57	GCA	4090	4147	58	GCA
Gene	coxI	3945	5522	1578	ATT/TAA	5349	6926	1578	ATT/TAA	4969	6546	1578	ATT/TAA	4150	5727	1578	ATT/TAA
Intergenic region		5523	5669	147		6927	7075	149		6547	6631	85		5728	5873	146	
Gene	nad4	5670	6929	1260	ATA/TAA	7076	8335	1260	ATA/TAA	6632	7891	1260	ATA/TAA	5874	7133	1260	ATA/TAA
tRNA	Thr	6900	6954	55	TGT	8306	8360	55	TGT	7862	7916	55	TGT	7104	7158	55	TGT
Gene	coxIII	6954	7721	768	ATT/TAG	8361	9128	768	ATT/TAA	7925	8695	771	TTG/TAA	7158	7925	768	ATT/TAG
tRNA	Leu	7722	7778	57	TAG	9129	9185	57	TAG	8693	8750	58	TAG	7926	7982	57	TAG
Gene	cytb	7785	8897	1113	ATT/TAA	9199	10,311	1113	ATT/TAA	8755	9867	1113	ATT/TAA	7989	9101	1113	ATT/TAA
tRNA	Phe	8898	8954	57	GAA	10,312	10,368	57	GAA	9868	9924	57	GAA	9102	9158	57	GAA
tRNA	Gln	8961	9015	55	TTG	10,375	10,430	56	TTG	9929	9983	55	TTG	9165	9219	55	TTG
tRNA	Arg	9017	9071	55	ACG	10,431	10,485	55	ACG	9984	10,038	55	ACG	9221	9275	55	ACG
tRNA	Ile	9072	9133	62	GAT	10,486	10,548	63	GAT	10,039	10,102	64	GAT	9276	9337	62	GAT
Gene	nad2	9133	9978	846	TTG/TAA	10,548	11,393	846	TTG/TAA	10,103	10,948	846	TTG/TAA	9337	10,182	846	TTG/TAA
tRNA	Ser	9979	10,033	55	TCT	11,394	11,448	55	TCT	10,949	11,003	55	TCT	10,183	10,237	55	TCT
tRNA	Leu	10,034	10,088	55	TAA	11,449	11,503	55	TAA	11,004	11,058	55	TAA	10,238	10,292	55	TAA
tRNA	Lys	10,089	10,150	62	TTT	11,504	11,565	62	TTT	11,059	11,120	62	TTT	10,293	10,354	62	TTT
Gene	atp6	10,153	10,752	600	ATT/TAA	11,568	12,170	603	ATT/TAA	11,124	11,723	600	ATT/TAA	10,357	10,956	600	ATT/TAA
Gene	nad1	10,755	11,630	876	TTG/TAA	12,172	13,047	876	TTG/TAA	11,725	12,600	876	TTG/TAA	10,959	11,834	876	TTG/TAA
tRNA	Tyr	11,628	11,684	57	GTA	13,045	13,101	57	GTA	12,598	12,653	56	GTA	11,832	11,888	57	GTA
tRNA	Asn	11,685	11,741	57	GTT	13,102	13,158	57	GTT	12,654	12,710	57	GTT	11,889	11,945	57	GTT
tRNA	Ser	11,742	11,795	54	Undef.	13,161	13,216	56	Undef.	12,711	12,778	68	TGA	11,946	11,999	54	Undef.
rRNA	rrnS	11,795	12,490	696		13,217	13,855	639		12,769	13,461	693		11,999	12,695	697	
tRNA	Glu	12,495	12,551	57	TTC	49	105	57	TTC	13,466	13,522	57	TTC	12,700	12,756	57	TTC
tRNA	Trp	12,554	12,610	57	TCA	107	163	57	TCA	13,525	13,581	57	TCA	12,759	12,815	57	TCA
Gene	nd4L	12,610	12,843	234	ATT/TAA	163	393	231	ATT/TAA	13,582	13,815	234	ATT/TAA	12,815	13,048	234	ATT/TAA
Gene	nad6	12,845	13,279	435	ATA/TAA	398	832	435	ATA/TAA	13,817	14,251	435	ATA/TAA	13,050	13,484	435	ATA/TAA
tRNA	Val	13,280	13,334	55	TAC	833	886	54	TAC	14,242	14,305	64	TAC	13,485	13,539	55	TAC
tRNA	Pro	13,335	13,388	54	TGG	887	940	54	TGG	14,306	14,360	55	TGG	13,540	13,593	54	TGG
AT‐rich region		13,389	13,766	378		942	1420	479		14,360	14,481	122		13,594	13,909	316	

Codon usage was very similar for the four species and the same preference toward one codon over other codons encoding for the same AA was present (Table 4). Only three codons were never used: CUC(L), CGC(R), and CGG(R). The ATT, ATA, and TTG start codons were used by all four species, while the ATG start codon was observed only once for the *nad5* gene in PmIII. The TAA stop codon was used for termination of all PCGs in PmII and PmIII, while the TAG stop codon was used once by PmI and PmIV for termination of the *coxIII* gene. Differences in the use of start and stop codons were observed between the four species in three genes: *nad5*,* coxII*, and *coxIII*. For *nad5*, PmIII used the start codon ATG, while the three other species used the ATT codon; for *coxII*, PmI and PmII used the start codon ATT, while PmIII and PmIV used the start codon ATA. For *coxIII*, PmI and PmIV had the same start and stop codon (ATT/TAG), PmII and PmIII used the same stop codon (TAA), but had different start codons, respectively ATT and TTG. For *coxII*, PmIII and PmIV used a different start codon (ATA), while PmI and PmII used ATT as start codon.

**Table 4:** Comparison of codon usage between the four cryptic species. Calculations are based on the 12 protein‐coding genes

Codon	PM1		PM2		PM3		PM4	
	Count	RSCU	Count	RSCU	Count	RSCU	Count	RSCU
UUU(F)	433	1.92	429	1.89	431	1.91	411	1.82
UUC(F)	19	0.08	25	0.11	20	0.09	40	0.18
UUA(L)	447	5.16	474	5.46	469	5.33	475	5.45
UUG(L)	40	0.46	28	0.32	22	0.25	15	0.17
CUU(L)	21	0.24	10	0.12	20	0.23	18	0.21
CUC(L)	0	0	0	0	0	0	0	0
CUA(L)	10	0.12	8	0.09	17	0.19	15	0.17
CUG(L)	2	0.02	1	0.01	0	0	0	0
AUU(I)	284	1.91	281	1.92	287	1.91	280	1.93
AUC(I)	13	0.09	11	0.08	14	0.09	10	0.07
AUA(M)	193	1.85	190	1.84	204	1.83	189	1.81
AUG(M)	16	0.15	16	0.16	19	0.17	20	0.19
GUU(V)	121	2.09	123	2.09	110	1.91	136	2.31
GUC(V)	6	0.1	1	0.02	3	0.05	5	0.08
GUA(V)	99	1.71	105	1.79	115	2	89	1.51
GUG(V)	6	0.1	6	0.1	2	0.03	6	0.1
UCU(S)	110	2.3	90	1.9	87	1.82	114	2.38
UCC(S)	1	0.02	3	0.06	0	0	2	0.04
UCA(S)	46	0.96	57	1.2	68	1.42	38	0.79
UCG(S)	0	0	3	0.06	2	0.04	1	0.02
CCU(P)	57	2.81	63	3.11	65	3.21	57	2.81
CCC(P)	10	0.49	6	0.3	0	0	6	0.3
CCA(P)	12	0.59	12	0.59	16	0.79	18	0.89
CCG(P)	2	0.1	0	0	0	0	0	0
ACU(T)	93	2.74	97	2.77	72	2.15	94	2.76
ACC(T)	4	0.12	0	0	3	0.09	3	0.09
ACA(T)	36	1.06	41	1.17	56	1.67	36	1.06
ACG(T)	3	0.09	2	0.06	3	0.09	3	0.09
GCU(A)	82	3.12	89	3.24	68	2.64	84	3.11
GCC(A)	3	0.11	2	0.07	7	0.27	3	0.11
GCA(A)	18	0.69	18	0.65	27	1.05	18	0.67
GCG(A)	2	0.08	1	0.04	1	0.04	3	0.11
UAU(Y)	156	1.91	154	1.93	159	1.94	153	1.88
UAC(Y)	7	0.09	6	0.07	5	0.06	10	0.12
UAA(*)	10	1.82	11	2	11	2	10	1.82
UAG(*)	1	0.18	0	0	0	0	1	0.18
CAU(H)	51	1.79	55	1.86	54	1.89	52	1.82
CAC(H)	6	0.21	4	0.14	3	0.11	5	0.18
CAA(Q)	41	1.78	35	1.52	39	1.73	34	1.48
CAG(Q)	5	0.22	11	0.48	6	0.27	12	0.52
AAU(N)	153	1.9	157	1.94	160	1.92	151	1.88
AAC(N)	8	0.1	5	0.06	7	0.08	10	0.12
AAA(K)	98	1.78	98	1.77	94	1.68	96	1.75
AAG(K)	12	0.22	13	0.23	18	0.32	14	0.25
GAU(D)	64	1.97	63	1.97	60	1.9	63	2
GAC(D)	1	0.03	1	0.03	3	0.1	0	0
GAA(E)	73	1.82	69	1.75	67	1.72	69	1.7
GAG(E)	7	0.17	10	0.25	11	0.28	12	0.3
UGU(C)	43	2	43	2	43	1.95	42	1.95
UGC(C)	0	0	0	0	1	0.05	1	0.05
UGA(W)	73	1.97	72	1.95	73	1.97	72	1.95
UGG(W)	1	0.03	2	0.05	1	0.03	2	0.05
CGU(R)	30	3.87	31	4	29	3.87	30	3.87
CGC(R)	0	0	0	0	0	0	0	0
CGA(R)	1	0.13	0	0	1	0.13	1	0.13
CGG(R)	0	0	0	0	0	0	0	0
AGU(S)	140	2.92	144	3.04	107	2.23	136	2.83
AGC(S)	8	0.17	3	0.06	4	0.08	7	0.15
AGA(S)	72	1.5	72	1.52	102	2.13	76	1.58
AGG(S)	6	0.13	7	0.15	13	0.27	10	0.21
GGU(G)	147	3.25	154	3.42	139	3.18	159	3.55
GGC(G)	1	0.02	4	0.09	2	0.05	1	0.02
GGA(G)	24	0.53	15	0.33	28	0.64	15	0.34
GGG(G)	9	0.2	7	0.16	6	0.14	4	0.09

RSCU: Relative Synonymous Codon Usage. In brackets, the coding amino acid is given.

P‐distance values based on all PCGs ranged between 6.1 and 10.5% within the *L. marina* species complex (Table 5). As a comparison, the P‐distance between *C. elegans* and *C. briggsae* was 13.9%, and values between *Litoditis* and *Caenorhabditis* ranged between 17.1 and 17.7%. The highest variability was found in the *nad* genes (Table 5).

**Table 5:** P‐distance values. Pairwise distance values were calculated for all protein‐coding genes, and as comparison, values between *Caenorhabditis elegans* and *C. briggsae* were calculated as well. Minimum and maximum distance percentage are shown. All ORFs is the value obtained from comparing all the 12 protein‐coding genes in one sequence each species

	Within *L. marina* complex		Within *Caenorhabditis*	Between *Litoditis* and *Caenorhabditis*	
	Min %	Max %	%	Min %	Max %
atp6	0.03	0.07	0.13	0.12	0.15
cytb	0.05	0.08	0.13	0.16	0.17
coxI	0.06	0.08	0.13	0.14	0.16
coxII	0.07	0.09	0.11	0.15	0.16
coxIII	0.07	0.10	0.13	0.15	0.19
nad1	0.07	0.12	0.16	0.16	0.19
nad2	0.06	0.13	0.15	0.22	0.25
nad3	0.06	0.14	0.19	0.16	0.22
nad4	0.07	0.14	0.14	0.17	0.20
nad4L	0.02	0.10	0.10	0.14	0.17
nad5	0.07	0.12	0.13	0.17	0.20
nad6	0.06	0.16	0.18	0.20	0.25
All ORFs	0.06	0.11	0.14	0.17	0.18

### Ribosomal and transfer RNA

The rRNAs were located on the same position for all four species: *rrnS* between tRNA^Glu^ and tRNA^Ser(Undef.)^ and *rrnL* between tRNA^His^ and *nad3*. Small differences in length were observed between the four species, ranging between 639 and 697 for *rrnS* and between 956 and 960 bp for *rrnL* (Table 3). The four mt genomes contained 22 tRNA genes. None of them had the conventional cloverleaf structure and showed a reduced T*ψ*C stem‐loop region. In all four species tRNA^Ser(TCT)^ lacked a D‐stem and had a reduced T*ψ*C stem, while only tRNA^Ser(TGA)^ of PmIII had a D‐stem and a T*ψ*C stem. For the other three species, the tRNA^Ser(TGA)^ could not be confirmed and its possible location was determined based on a BLASTn search with *C. elegans* as a query. This tRNA is named tRNA^Ser(Undef.)^, due to the unknown anticodon. Some tRNAs had overlap of 1–30 nt with adjacent genes. In all four species the largest overlap was observed for tRNA^Thr^ with 30 nucleotides, followed by an overlap of 3 nt for tRNA^Tyr^. PmI, PmII, and PmIV also had an overlap of tRNA^Ile^ and tRNA^Trp^ in common with 1 nt. Overlap in tRNA^Ser(Undef.)^ with 1 nt was found in PmI and PmIV. PmIII had an overlap of 3 nt and 10 nt for tRNA^Leu(TAG)^ and tRNA^Ser(TGA)^, respectively.

### Noncoding regions

The AT‐rich region is a highly variable region with 310 variable positions in a 958‐bp‐long alignment. It is located between tRNA^Pro^ and tRNA^Ala^ in all four species, but showed pronounced differences in length for species PmIII compared to the three other species (PmI: 378 bp; PmII: 479 bp; PmIII: 953 bp; and PmIV: 543 bp).

An intergenic region located between *coxI* and *nad4* in all four species is also highly variable (45 positions/149 bp). The length of the intergenic region was similar for PmI, PmII, and PmIV, being respectively 147, 149, and 146 bp long. PmIII was different in that it had two intergenic regions, one located between *coxI* and *nad4* like the other three species but considerably shorter (85 bp long), and one (206 bp long) located between tRNA^Met^ and tRNA^Asp^.

### Phylogenetic relationships and timing of divergence

**Figure 2:**
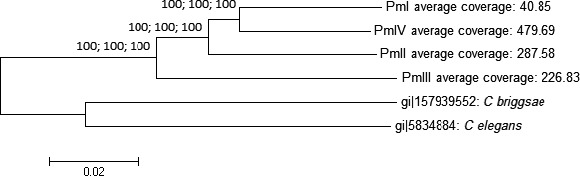
Phylogenetic relationship based on neighbor joining of the four cryptic *Litoditis* species. Nodes show bootstrap values of, from left to right, maximum likelihood, neighbor‐joining and maximum parsimony analysis. *Caenorhabditis elegans* and *C. briggsae* are used as outgroup taxa.

**Figure 3:**
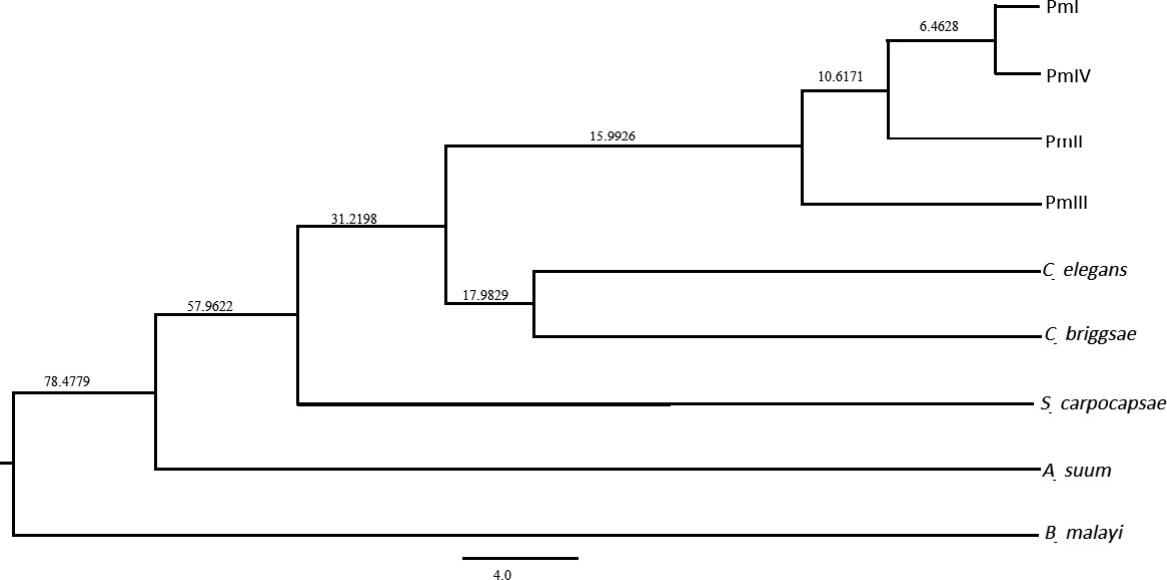
Timing of the most recent common ancestor. The divergence time of *C. elegans* and *C. briggsae* 18 MYA was used as calibration point, based on mutation rates (Cutter 2001).

The PCG alignment was 10380 bp long. The *Litoditis marina* complex contained 1631 variable and 261 parsimony‐informative positions. Maximum likelihood, neighbor‐joining, and maximum parsimony trees gave the same topology, and each branch was supported by bootstrap values of 100. PmI and PmIV were sister taxa, while PmIII was most distantly related to the three other species (Fig. 2). The time tree calculated in BEAST (Fig. 3) showed that the most recent common ancestor for the *Litoditis marina* species complex was situated at 16 million years ago (MYA). PmII diverged 10.5 MYA from PmI and PmIV, while the latter two diverged 6.5 MYA. Time calculations were the same when using one or two calibration points.

### *Wolbachia* detection

A BLASTn search was performed with the *Wolbachia* sequences as queries against the complete genomes of the four cryptic species, containing both nuclear and mtDNA. Of the five conserved genes of the Multilocus Sequence Typing system (MLST), two genes showed no significant match, while the three other genes were found with a low % identity (<75%). Also the *Wolbachia* surface protein (*wsp*) and the 16S RNA gene were not detected in the sequence data of the four species. These results suggest that *Wolbachia* was not present in the genome assemblies of these species.

### Mode of selection on PCGs

**Figure 4:**
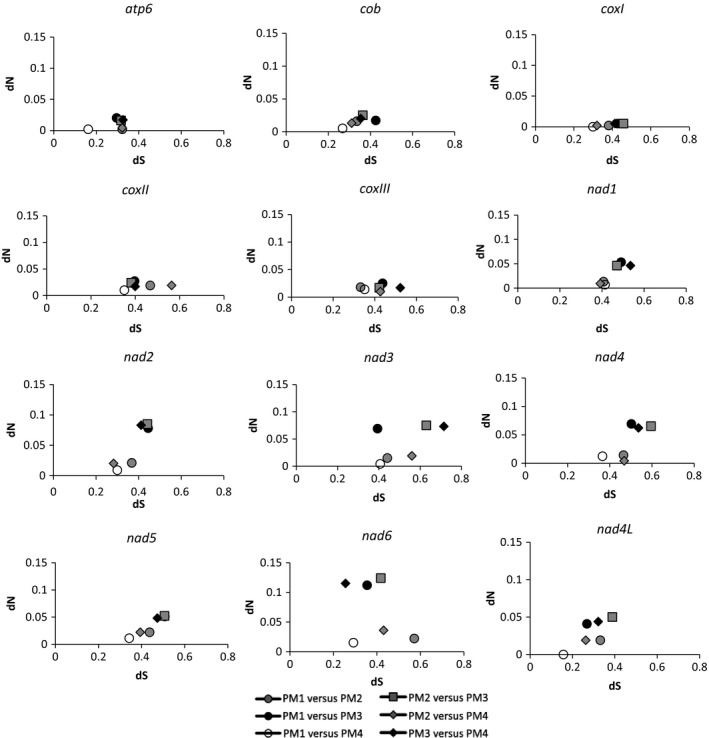
Scatter plots of nonsynonymous (dN) to synonymous (dS) nucleotide substitutions in the protein‐coding genes between all pairwise comparisons of the *Litoditis marina* species. All PCGs were evolving under negative (purifying) selection (dN/dS < 1). Colors of symbols reflect degree of relatedness (white: recent sister species; gray: intermediate timing of relationship between species; black: oldest relationships between species).

The ratio of nonsynonymous (dN) to synonymous (dS) substitutions of the PCGs were significantly larger than zero and smaller than 1, suggesting that all genes were experiencing purifying selection. The Complex V gene (*atp6*), the Complex III gene (*cob*) and the Complex IV genes (*coxI‐III*) were under strongest selection, while the Complex I genes (*nad1‐6*) were under weaker selection. These differences in strength of selection are reflected in the near horizontal relationship between dN and dS for the genes under strong selection (*atp6, cob, coxI‐III*) and in a diagonal relationship for the genes under weaker selection (Fig. 4). The short genes from the Complex I genes (*nad3* and *nad6*) exhibited a more scattered pattern. The Complex I genes had a higher synonymous substitution rate, with dS‐values exceeding 0.5. Finally, the closest related species (PmI and PmIV) clearly accumulated less nonsynonymous mutations compared to the more distantly related species (Fig. 4).

## Discussion

Free‐living marine nematodes are characterized by a very high global species diversity, and nematode assemblages typically show high local diversity (Heip et al. 1985). Much work has focused on interactions among species and between species and their environment, and on the importance of nematodes for the functioning of marine benthic systems (Nascimento et al. 2012), yet only little is known on the evolutionary processes that have mediated this diversity. In addition, the presence of cryptic species may substantially complicate patterns of diversity. In marine nematodes, cryptic species have been detected based on diverging lineages in the mitochondrial COI gene and ribosomal nuclear genes (Derycke et al. 2005, 2007), and subsequent studies have demonstrated that these cryptic species show subtle morphological differentiation (Derycke et al. 2008a,b, 2010), reproductive isolation (Fonseca et al. 2008), and ecological differences (De Meester et al. 2011, 2012a,b; Van Campenhout et al. 2014). Our results now demonstrate high synteny in the mitochondrial genomes of the four species, but differences in gene length, in number of intergenic regions, and in the use of start/stop codons between some species.

### High synteny in the mitochondria of the *L. marina* cryptic species complex

A survey in 62 nematode mitochondrial genomes, of which 54 species belong to the same order as our *Litoditis marina* species, revealed 25 different gene arrangement patterns (Liu et al. 2013). Variability in gene order is often due to a different location of the tRNA genes (Gissi et al. 2008), but this was not the case for the four *Litoditis* species. The gene order in the mitochondrial genomes of all four cryptic species was identical and corresponds to the typical gene order of several other rhabditid nematodes (Sun et al. 2014). The four mitochondrial genomes were highly AT‐rich and codon usage was biased toward AT‐rich codons, a pattern that has been frequently observed in Nematoda (Rota‐Stabelli et al. 2010). The use of start and stop codons was highly comparable to that observed in other Rhabditina (Hu et al. 2002), including *Caenorhabditis elegans* (Okimoto et al. 1992), but different usage between the *Litoditis* group and *C. elegans* was observed for the *nad4*,* cob,* and *nad1* gene. Furthermore, the use of incomplete stop codons such as T and TA has frequently been observed in Nematoda (Okimoto et al. 1992; Hu et al. 2002; Liu et al. 2013), but this was not the case for the *Litoditis* species. The use of the ATG start codon by PmIII for the initiation of the *nad5* gene is unusual for metazoan mitochondria, but has also been reported in *Ancylostoma duodenale* (Hu et al. 2002), also a member of the Rhabditina. Transfer RNA in nematodes very often lacks one or two arms and shows replacement loops instead (He et al. 2005; Jühling et al. 2012). This was also observed for the *L. marina* species complex, where twenty tRNAs had a replacement T stem‐loop, and the two tRNA^Ser^ had a replacement D stem‐loop. The annotated tRNAs further showed an overlap with the adjacent genes. Although overlaps up to 14 nucleotides have been recorded in enoplean nematodes (Jühling et al. 2012), the largest overlap in *Litoditis* was tRNA^Thr^ with 30 nucleotides. Overlapping tRNAs can cause problems in tRNA processing, because they have a specific tRNA precursor and the upstream tRNA can miss the overlapping nucleotides (Reichert et al. 1998; Mörl and Marchfelder 2001). The ribosomal RNAs of the *Litoditis* species showed only minor differences in length, and was comparable to the length of the ribosomal RNAs of *C. elegans* (697 and 953 bp, respectively) and of other nematodes (Hu et al. 2002).

The mitochondrial genomes of all four species lack the *atp8* gene, which encodes for a core subunit of the F0 domain of the ATPase (da Fonseca et al. 2008). The absence of the *atp8* gene has been observed in other nematodes as well (Okimoto et al. 1992; Montiel et al. 2006; Sultana et al. 2013; Leung et al. 2013), while a putative form has been found in the nematode *Trichinella spiralis* (Lavrov and Brown 2001). Genes encoding for the ATPase complex show a tendency to be lost in the fast‐evolving metazoan mtDNA (Gissi et al. 2008). Despite the high synteny in the mitochondrial genomes of the four cryptic species, our mitogenomic approach also revealed differences in genome length, gene length, nucleotide overlap between adjacent genes, and start and stop codon usage mostly between PmIII and the three other species.

### The mitochondrial genome of PmIII shows several differences compared to PmI, PmII, and PmIV

Differences in mitochondrial structural organization may have strong impacts on the physiology of the organism because mitochondria have an important role in energy metabolism. It was recently shown that a single nonsynonymous amino acid change in the mitochondrial cytochrome oxidase c subunit 1 (*coxI*) gene of geographical isolates of *C. elegans* substantially affects their longevity when exposed to different temperatures, which suggests that mitochondrial energy metabolism may be critical to adapt to environmental changes related to temperature (Dingley et al. 2014). Although we lack information on the longevity of these four species, recent experimental work shows that PmIII has a higher instantaneous fecundity than the other three species and performs better at higher temperature (De Meester et al. 2015). The *coxI* gene of the four species shows four nonsynonymous amino acid changes, three of which occur between PmIII and the three other species. PmIII further contains a substantially longer *nad3* gene, which is part of the electron transport chain in Complex I (da Fonseca et al. 2008). Depending on their location and size, insertions and deletions can significantly alter the structure and function of proteins, and may be related to adaptation to particular environmental conditions (Wang et al. 2009). It is therefore possible that the structural differences in mitochondria may, at least in part, explain the biological differences between some cryptic species.

### Phylogeny of the PCGs supports PmI and PmIV as sister taxa and shows a Miocene origin for the *Litoditis marina* species complex

The phylogenetic relationships obtained with the PCGs are consistent with previous published phylogenies of the *Litoditis* species complex based on nuclear data (Derycke et al. 2005, 2008a,b) and recovered PmI and PmIV as sister taxa. Although relationships based only on the *coxI* gene have put PmII forward as the earliest diverging species (Derycke et al. 2005), our mitogenomic approach now supports PmIII as the earliest diverged species. This is in agreement with the large number of structural differences observed in the mitochondria of PmIII and with the high similarity in mitochondrial structure of PmI and PmIV. The presence of *Wolbachia* could affect the mutation rate and fixation of mutations due to sweeps in the mtDNA (e.g., Shoemaker et al. 2004) and can in this way affect phylogenies. In nematodes, *Wolbachia* is commonly found in filarial nematodes, but they have not been observed in secernentean nematodes (Bordenstein et al. 2003). Our data further support the lack of *Wolbachia* in *Litoditis*, a secernentean genus.

The most recent common ancestor for the *Litoditis marina* species complex was estimated at 16 MYA during the Miocene. PmII diverged 10.5 MYA from PmI and PmIV, while the latter two diverged 6.5 MYA. These dates are much older than the last glaciations and tectonic activities in the Atlantic Ocean with the formation of the North Atlantic–Arctic gateway and the Mediterranean Sea (Stoker et al. 2002; Harzhauser and Piller 2007), but they cannot be linked to mass extinction or other well‐known geographical events. These old speciation events clearly show that cryptic species are not necessarily of recent origin. It is therefore unlikely that their similar morphology is caused by a lack of sufficient time to incorporate morphological differences. Morphological changes among species are low when strong selection on behavioral or physiological characteristics for adaptation to a specific host (Schonrogge et al. 2002) or environment are required. The *Litoditis* species thrive on decomposing algae in the intertidal environmental, where they are exposed to strong fluctuations in temperature and salinity. The ephemeral nature of the algal habitat requires the ability to rapidly colonize and reproduce to quickly establish viable populations. *Litoditis* is thus very likely to be subjected to strong environmental pressures. Visual recognition between species is often hampered in the marine environment and more pressure toward physiological distinctions (e.g., chemical characteristics) is likely to be important. We found evidence for purifying selection on all mitochondrial PCGs. Purifying selection is necessary for mitochondrial genes to maintain function (Rand 2001), although there are some reports of positive selection as well (summarized in (Castellana et al. 2011)). The *coxI*‐*III*,* cob,* and *atp6* genes are highly conserved in all four species, with strong purifying selection. The more closely related the species are, the higher the conservation in the genes between the species. The *nad1*‐*6* genes show higher variation with dS‐values exceeding 0.5 in the genes with short sequences (*nad3* and *nad6*). These genes have a higher rate of mutational saturation, which may lead to inaccurate dN/dS ratios. An earlier study in which dN/dS ratios were estimated from 347 complete vertebrate mt genomes showed that purifying selection was strongest for genes that encode subunits with crucial functions in the respiratory chain (RC), for example, *cob* and the *coxI*‐*III* genes (Castellana et al. 2011). It has been put forward that the accumulation of non‐neutral mitochondrial genetic variation within populations might play a role in speciation, with negative selection on mito‐nuclear interactions (Dowling et al. 2008). These mito‐nuclear interactions result in coevolution, with mtDNA mutations acting as drivers of adaptations in the nuclear genome (Dowling et al. 2008).

## Conclusion

Despite millions of years of evolution, the mitochondrial genomes of the four cryptic *Litoditis marina* species show no variation in gene order or in gene content. However, many synonymous mutations have occurred, and structural differences between some species were present. Speciation of the cryptic species was estimated to have occurred in the Miocene, illustrating that the morphological similarity is not caused by insufficient time to evolve. Instead, differences in mitochondrial genome structures have accumulated between the earliest diverged species (PmIII) and the other three species, which may be linked to the different environmental adaptations observed in these species.

## Conflict of Interest

None declared.
